# Editorial: Phage-based strategies for foodborne pathogen mitigation and detection

**DOI:** 10.3389/fmicb.2025.1649376

**Published:** 2025-07-08

**Authors:** Aneta Skaradzińska, Bartłomiej Grygorcewicz, Paulina Śliwka, Robert Czajkowski

**Affiliations:** ^1^Department of Biotechnology and Food Microbiology, Faculty of Biotechnology and Food Science, Wrocław University of Environmental and Life Sciences, Wrocław, Poland; ^2^Department of Genomics and Forensic Genetics, Pomeranian Medical University in Szczecin, Szczecin, Poland; ^3^Regional Center for Digital Medicine, Pomeranian Medical University in Szczecin, Szczecin, Poland; ^4^Laboratory of Biologically Active Compounds, Intercollegiate Faculty of Biotechnology of the University of Gdańsk and Medical University of Gdańsk, University of Gdańsk, Gdańsk, Poland

**Keywords:** bacteriophage, phage application, food safety, foodborne illness, foodborne pathogens

The global burden of foodborne illnesses remains a critical public health challenge. According to the World Health Organization (WHO), the consumption of unsafe food results in approximately 600 million cases of foodborne illness and 420,000 deaths worldwide each year. Alarmingly, children under the age of five account for nearly 30% of these fatalities. Furthermore, WHO estimates that the global burden of unsafe food corresponds to a loss of 33 million healthy life years annually—a figure that is likely underestimated (World Health Organization, 2025).

Notably, food contamination with pathogenic microorganisms not only leads to significant morbidity and mortality but also imposes substantial economic losses throughout the food production and supply chain. In light of increasing consumer demand for safer, minimally processed, and antibiotic-free food products, the development of alternative antimicrobial strategies has become imperative. Among the most promising options is the application of bacteriophages. The aim of this Research Topic was to bring together cutting-edge studies and perspectives regarding the applications of phages in food safety.

Infections caused by *Salmonella enterica* are among the most common types of foodborne illness. Transmission typically occurs through the consumption of contaminated food, especially raw or undercooked meat, eggs, or unpasteurized dairy products. Symptoms include diarrhea, fever, abdominal cramps, and vomiting, which can be particularly severe in young children, the elderly, and immunocompromised individuals. In some cases, the infection can lead to life-threatening complications such as sepsis. Of particular concern is the emergence of antibiotic-resistant *Salmonella* strains, which significantly complicate treatment and highlight the urgent need for novel antimicrobial agents (Eng et al., 2015).

Jaglan et al. isolated a broad-host-range lytic phage, phiSalP219, effective against both typhoidal and non-typhoidal *S. enterica*. The phage showed resistance to low pH and high temperatures, lacked virulence and resistance genes, and remained stable on food surfaces like salami and chicken ham. It also effectively disrupted biofilms, leaving only cellular debris. The potential of phages as antibiofilm agents should be underscored. Biofilms confer high resistance to conventional antimicrobials and disinfectants and pose risks at nearly every stage of food production (Sharma et al., 2023).

The antibiofilm properties of phages were also confirmed by Ma et al., who demonstrated that phage SPB, targeting methicillin-resistant *Staphylococcus aureus* (MRSA), inhibited both planktonic bacterial growth and biofilm formation. Phage SPB significantly suppressed biofilm development and eradicated established biofilms. *S. aureus* is considered a high-risk pathogen due to its wide array of virulence factors, which contribute to its ability to cause various human infections, including those transmitted through contaminated food. MRSA strains raise particular concern due to their antibiotic resistance (Merghni et al., 2023).

Another foodborne pathogen of major concern is *Escherichia coli*. Although most strains are harmless, certain types, such as *E. coli* O157:H7, can cause severe gastrointestinal illness, including bloody diarrhea, abdominal cramps, and vomiting. Infections are commonly linked to contaminated food or water, especially undercooked ground beef, raw vegetables, and unpasteurized dairy products (Gambushe et al., 2022).

Yesil et al. characterized phage OSYSP, with activity against both *E. coli* and *S. enterica*. Notably, this phage was highly effective against three *E. coli* O157:H7 strains and a non-pathogenic *E. coli* K12 strain, although its activity against *Salmonella* strains was limited. The authors noted that interactions with non-target bacteria might reduce OSYSP's efficacy in clinical settings. Nevertheless, its robustness—evidenced by 2 years of stability in cold storage and resilience under various environmental conditions—makes it a strong candidate for practical use.

Kim E. et al. also investigated phage-based control of *E. coli* in food systems. Two newly isolated phages, LEC2 and LEC10, demonstrated a broad host range, lysing approximately 80% of the tested *E. coli* strains, including those isolated from clinical cases. The complementary host ranges of LEC2 and LEC10 led to the development of a phage cocktail, which exhibited superior bactericidal activity against mixed *E. coli* populations compared to individual phages. Furthermore, the cocktail significantly reduced *E. coli* counts in naturally contaminated fresh vegetables, demonstrating efficacy beyond laboratory conditions.

The potential of phage cocktails was further explored by Hammerl et al., who proposed a combination of three novel phages—vB_YenM_P8, vB_YenM_P744, and vB_YenM_P778—to target *Yersinia enterocolitica*, a psychrotrophic foodborne pathogen of clinical relevance. This bacterium can survive and multiply at refrigeration temperatures, making it particularly problematic in cold-stored foods such as pork, milk products, and ready-to-eat meals. *Y. enterocolitica* infections (yersiniosis) can cause gastrointestinal symptoms and, in severe cases, complications like reactive arthritis (Li et al., 2020). The three phages lysed all 53 tested *Y. enterocolitica* strains. Combined with previously characterized phages vB_YenM_P281 and vB_YenP_Rambo, the cocktail showed strong lytic activity across serotypes and temperatures, suggesting efficacy under food-processing conditions. Genetic analyses showed the phages were unrelated and had different host specificities at various temperatures, suggesting they likely bind to distinct bacterial receptors. This diversity reduces the risk of resistance development and enhances the cocktail's effectiveness.

A similar receptor-targeting approach was taken by Kim S. et al., who developed a cocktail of two phages—CR8 and S13—against *Cronobacter sakazakii*. CR8 uses bacterial flagella for attachment, while S13 targets lipopolysaccharide (LPS) structures. *C. sakazakii* is an opportunistic foodborne pathogen particularly dangerous to neonates and immunocompromised individuals. It is primarily associated with contaminated powdered infant formula and can lead to severe outcomes such as meningitis or sepsis (Mardaneh, 2021). The phage cocktail displayed enhanced lytic activity and more effectively delayed resistance emergence compared to individual phages, underscoring the potential of multi-receptor-targeting phage combinations.

In conclusion, this Research Topic confirms that phage-based approaches are no longer a niche research topic but are becoming a mainstream component of food microbiology and safety. From farm to fork, phage tools can be integrated into modern food systems and support global initiatives to reduce antimicrobial resistance. As research continues to uncover their full potential and optimize their application, bacteriophages stand out as a safe, effective, and environmentally friendly solution in the fight against foodborne pathogens. Together, these advancements signal a promising future for phage-based innovations in ensuring global food safety.
